# Epidemiology and clinical outcomes of advanced maternal age: a cross-sectional study at Saint-Joseph Hospital and the University Clinics of Kinshasa in the Democratic Republic of Congo

**DOI:** 10.11604/pamj.2025.51.49.47389

**Published:** 2025-06-17

**Authors:** Antoine Tshimbundu Kayembe, Anne Kapinga Mutshiaudi, Alex Mutombo Baleka, Andy Mbangama Muela, Rahma Raschid Tozin, Roger Mbungu Mwimba, Dieudonné Sengeyi Mushengezi, Patrick Kahindo Muyayalo

**Affiliations:** 1 Department of Gynaecology and Obstetrics, Faculty of Medicine, University Notre-Dame of Kasayi, Central Kasaï, Democratic Republic of Congo,; 2 Department of Gynaecology and Obstetrics, Faculty of Medicine, University of Kinshasa, Kinshasa, Democratic Republic of Congo

**Keywords:** Epidemiology, clinical, advanced maternal age, Saint-Joseph Hospital, university clinics, Kinshasa

## Abstract

**Introduction:**

advanced maternal age is defined as women aged 35 years or older at the estimated date of delivery based on the woman's physiology. Pregnancies at this maternal age are at risk for adverse obstetric outcomes. The objective of this study is to determine the epidemiological and clinical profile of elderly mothers at Saint-Joseph Hospital and the University Clinics of Kinshasa in the Democratic Republic of Congo.

**Methods:**

this is a cross-sectional study of a series of records of elderly mothers who were followed and gave birth in the maternity wards of Saint-Joseph Hospital and the University Clinics of Kinshasa from January 1, 2018, to December 31, 2022, using non-probability convenience sampling for case selection. Descriptive analyses were performed in the statistical analyses.

**Results:**

the birth rate among advanced mothers was 28.7% with their average age of 38.65 (SD: 2.64) years, housewives, married mothers and those with university education were present respectively in 62.78%, 95% and 49.75% of cases, their average parity was 3.69 (SD: 1.96) deliveries and primiparous mothers were present in 43.2% of cases. The spontaneous abortion´s history was present in 43.2% of cases, scarred uterus history in 29.61% of cases, pregnancy-induced hypertension´s history in 6.1%, and pre-eclampsia´s history in 2.53% of cases. The advanced maternal age was significantly associated with the occurrence of pre-eclampsia (aOR: 3.5, 95% CI: 1.3-9.5, p=0.002), pregnancy-induced hypertension (aOR: 2.3, 95% CI: 1.7-7.8, p=0.012), prematurity (aOR: 2.7, 95% CI: 1.4-5.0, p=0.001), low birth weight (aOR: 2.5, 95% CI: 1.1-63, p=0.002) and admission to neonatal unit (aOR: 2.5, 95% CI: 1.0-6.3, p=0.020).

**Conclusion:**

the pregnancy in mothers with advanced maternal age is a real public health problem, primiparas with university education, married women, and housewives with a history of spontaneous abortion and scarred uterus are more affected. Adverse obstetric outcomes include preeclampsia, pregnancy-induced hypertension, prematurity, low birth weight, and admission to the neonatal unit.

## Introduction

Advanced maternal age (AMA) is defined as women aged 35 years or older at the estimated date of delivery, based on the woman's physiology [1,2]. However, some authors consider “late” pregnancy to be that occurring after age 40, due to a higher risk of obstetric complications [1]. Pregnancies in women of advanced maternal age are pregnancies at high risk of maternal-fetal morbidity and mortality [2-4]. This risk is increased by age-related physiological and physical changes [5]. Patients of advanced age have more comorbidities, such as type 2 diabetes and chronic hypertension, in addition to the morbid history of pregnant women, such as uterine scars from myomectomy or cesarean section [5]. The global birth prevalence among mothers of advanced age was 12.3% [6,7]. In Canada, the prevalence of women with AMA increased from 15% in 1998 to 18% in 2007, and 1 in 3 primiparas was over 35 years old [8]. In the USA, the reproductive age increased from the early twenties in 1970 to the late twenties in 2006 [8]. In France, this prevalence increased from 19% in 2010 to 21% in 2016 (8). These prevalences also increased from 8.6% in 1990 to 25.9% in 2012 in Japan [3]. Similar trends have been observed in other developed countries, in England [9,10] and Australia [11].

In Asia, the birth prevalence among women in AMA varies from 3 to 31% and Japan has the highest birth prevalence among mothers in AMA (which is 31.0%) in the world [3]. In Africa, the birth prevalence among mothers of advanced age varies from 8.0% to 18.0% [3]. Several African studies have reported a delay in the age at first pregnancy: this is the case in Cameroon in 2022 and Tanzania in 2017 [3,6]. In the Democratic Republic of Congo (DR Congo), this prevalence is 18.0%, the highest on the African continent [3].

The spread of schooling with the extension of the duration of studies, family planning, and today the advent of assisted reproductive technology (ART) are associated with the evolution of this birth prevalence among women in AMA in several studies [8]. A hospital study conducted by Kaka *et al*. found a birth rate of 14.65% in 2014 in Kinshasa, and predicted the increase in the frequency figure [12]. What is the epidemiological situation of mothers in AMA 10 years later in our city? The answer to this previous question justifies the initiation of this study.

The objective of this study is to determine the epidemiological and clinical profile of elderly mothers at Saint-Joseph Hospital (HSJ) and the University Clinics of Kinshasa (UCK) in DR Congo.

## Methods

**Study design and setting:** this is a cross-sectional study of a series of records of elderly mothers (over 35 years old) followed and who gave birth in the maternity wards of two hospitals in the city of Kinshasa: HSJ and UCK, from January 1, 2018, to December 31, 2022. These two hospitals were chosen because of the presence of specialists in the fields of gynecology and obstetrics, which attract a high number of women from the city of Kinshasa, and the existence of a technical platform worthy of second-referral hospitals for HSJ and third-referral hospitals for UCK.

**Study population:** our study population consists of records of elderly mothers (over 35 years old) who were followed and gave birth in the maternity wards of two hospitals in the city of Kinshasa: HSJ and UCK, from January 1, 2018, to December 31, 2022. Our sampling is non-probability-based on convenience. The sample size was dictated by the limitations of our study in time and space. The following criteria allowed us to include patients in the study: records of mothers over 35 years old who attended prenatal consultations and delivered a preterm (28 to 36 weeks) and term (over 36 weeks) newborn in the Obstetrics Department of the UCK and at the HSJ during the period of our study. We excluded from this study the records of women of advanced maternal age who gave birth before 28 weeks of gestation, those that were incomplete, and those that were not found in the same setting and during the same study period. After applying our selection criteria, we retained a total of 2,285 records of women undergoing AMA for this study. We note that 836 records were excluded from this study for various reasons, including delivery before 28 weeks of gestation (555 records), and records that were not found or incomplete (281 records).

**Data collection:** data were collected by searching the medical records of the women who gave birth and the registers of the maternity and operating rooms of these two hospitals, and recorded in the data collection record. The variables in our study were: year, age of the women who delivered, sociodemographic characteristics such as occupation, marital status, level of education, and clinical characteristics such as parity, gestational age, history of abortion, smoking, diabetes, high blood pressure, scarred uterus, preeclampsia, and pregnancy-induced hypertension, type of pregnancy and delivery and adverse perinatal and maternal outcomes. Our data were collected as follows: we first identified the names of the women who delivered included in the study from the antenatal, maternity, and operating room registers of HSJ and of UCK, then searched medical records based on the names retained from the registers. Once the records were located, the data from the records was transcribed onto the data collection sheet.

**Operational definition:** parity is the number of deliveries. A primiparous woman is defined as a woman who has already given birth once, a pauciparous woman is a woman who has already given birth two to three times, a multiparous woman is a woman who has already given birth four to six times, and a grand multiparous woman is a woman who has already given birth seven times or more [12]. Abortion: this is the spontaneous or voluntary termination of a pregnancy before the age of viability [12].

**Statistical analysis:** data were entered using Excel and exported to SPSS version 29 software for statistical analysis. Quantitative variables were presented as mean (standard deviation), and qualitative variables as proportions. We used ANOVA tests to compare means, chi-square tests to compare proportions, and univariate logistic regression to assess the strength of association between obstetric outcomes and AMA. Odds ratios (OR) were adjusted for parity, history of hypertension, and education level. The significance of our results was set at P < 0.05. The variables that were statistically significantly associated with pregnancy from women with AMA in the ANOVA test and Chi^2^ test were included in the univariable model, while those that were significantly associated with pregnancy from women with AMA in the univariable model were included in the multivariable model.

**Ethical considerations:** the research protocol for this study was approved by the staff of the Department of Gynecology and Obstetrics of UCK and HSJ, acting as the Medical Ethics Committee. The reference number of the approval by the Ethics Committee is N°015/KMP/GO/2023. The principles of medical ethics and the rules for documentary studies were respected: data were collected confidentially and treated anonymously.

## Results

**Figure 1:**
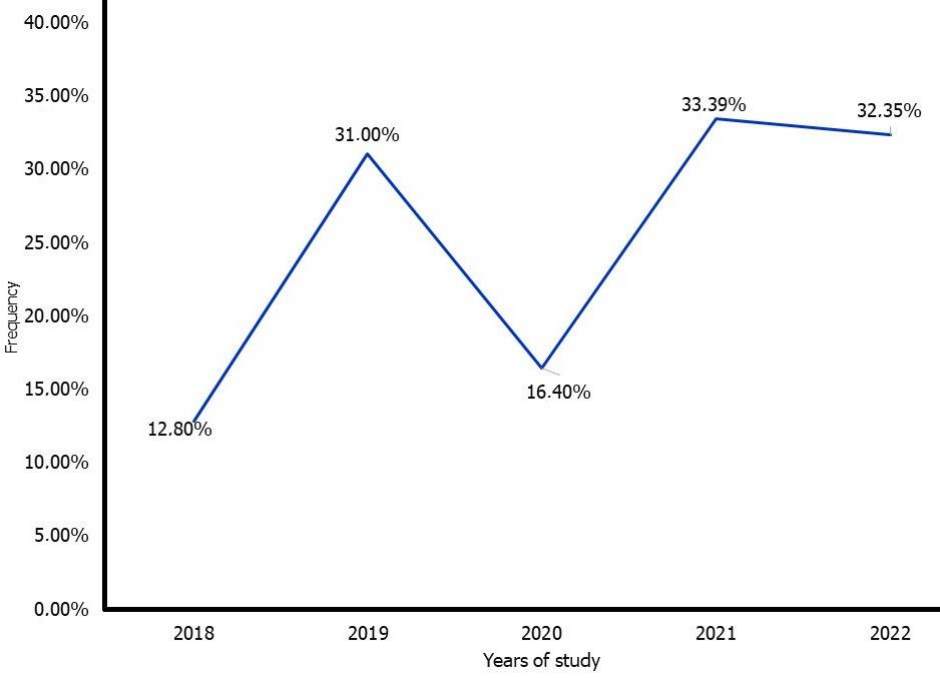
evolution of advanced maternal age (AMA) frequency during the study period

**Birth rates among advanced mothers:** during our study period, 10,878 deliveries were recorded in the UCK and HSJ maternity units, of which 3,121 were from women with AMA, representing a birth rate among advanced mothers of 28.7% during our study period. This birth rate ranged from 12.80% in 2018 to 32.35% in 2022, passing through 31.00% in 2019, 16.4% in 2020, and 33.90% in 2021 during our study period (Figure 1). We note that 836 records were excluded from this study for various reasons, including deliveries before 28 weeks of amenorrhea (555 records) and records that were not found or were incomplete (281 records).

**Sociodemographic characteristics of AMA:** the average age of advanced mothers was 38.65 (SD: 2.64) years, with 73.60% of mothers between the ages of 35 and 39. Housewives were present in 62.78% of cases, married women in 95% of cases, and university-educated women in 49.75% of cases (Table 1).

**Table 1:** distribution of cases according to socio-demographic and clinical characteristics

Variables	n=2285	%*
Age of mothers in AMA	38.65 (SD: 2.64)	
35-39 years	1682	73.6
≥40 years	603	26.4
**Profession**		
Commerçante	119	5.25
Student	43	1.9
Fonctionnaire	253	11.09
Housewife	1434	62.78
Medical personnel	301	13.20
No formal employment	131	5.77
**Marital status**		
Single	89	3.9
Married	2285	95
Widowed	25	1.10
**Level of education**		
None	46	2.0
Primary	45	1.97
Secondary	1057	46.26
University	1137	49.75
Parity, mean (SD)*	3.69 (1.96)	
Primiparity	987	43.2
Pauciparity	820	35.9
Multiparity	478	20.9
Gestity, mean (SD)*	4.4 (SD: 2.21)	
History of abortion	985	43.10
1 - 2	819	35.84
≥ 3	166	7.25
History of uterine scarring	677	29.61
History of preeclampsia	58	2.53
History of pregnacy-induced hypertension	139	6.1
History of diabetes	29	1.27
History of hypertension	11	0.49

* SD: standard deviation; ≥: superior or aqual to; %: percentage; AMA: advanced maternal age

**Clinical characteristics of AMA:** the average parity of women with AMA was 3.69 (SD: 1.96) deliveries overall, with an average parity of 4.14 (SD: 2.08) deliveries for the over-40 age group and 3.23 (SD: 1.83) deliveries for the 35-39 age group. Primiparous women were present in 43.2% of cases, pauciparous women in 35.9%, and multiparous women in 20.9% of cases. The average gestation was 4.4 (SD: 2.21) pregnancies in general with the gestation of 4.77 (SD: 2.41) pregnancies for the group over 40 years and 4.03 (SD: 2.01) for the group of 35 to 39 years, the history of abortions was present in 43.10% of cases with 1 to 2 abortions in 35.84% of cases and more than 3 abortions in 7.25% of cases, those of scarred uterus were present in 29.61% of cases, those of pre-eclampsia (PE) in 2.53% of cases, those of diabetes mellitus (DM) in 1.27% of cases and those of gestational hypertension or of pregnacy-induced hypertension in 6.10% of cases (Table 1).

Regarding adverse obstetric outcomes, advanced maternal age was significantly associated with the occurrence of pre-eclampsia (aOR: 3.5, 95% CI: 1.3-9.5, p=0.002), pregnancy-induced hypertension (aOR: 2.3, 95% CI: 1.7-7.8, p=0.012), prematurity (aOR: 2.7, 95% CI: 1.4-5.0, p=0.001), low birth weight (aOR: 2.5, 95% CI: 1.1-63, p=0.002) and admission to neonatal unit (aOR: 2.5, 95% CI: 1.0-6.3, p=0.020) (Table 2).

**Table 2:** univariable and multivariable analyses

	Univariable analysis		Multivariable analysis	
Adverse obstetric outcomes	aOR (95% CI)	P-Value	aOR (95% CI)	P-Value
Preeclampsia	2.5 (1.3-5.0)	0.001	3.5 (1.3-9.5)	0.002
Pregnancy-induced hypertension	5.3 (2.1-13.8)	0.001	2.3 (1.7-7.8)	0.012
Prematurity	2.3 (1.3-4.0)	0.001	2.7 (1.4-5.0)	0.001
Low birth weight	1.9 (1.0-3.8)	0.001	2.5 (1.1-5.6)	0.002
Admission in neonatal unity	2.5 (1.7-4.4)	0.001	2.5 (1.0-6.3)	0.020
Neonatal death	2.6 (1.2-5.8)	0.001	1.6 (0.4-6.6)	0.110

aOR: ajusted odd-ratio; 95% CI: 95% confidence interval; P-value: the significant p-value set to less than 0.05

## Discussion

This study aimed to determine the epidemiological and clinical profile of elderly mothers at the HSJ and UCK in the DR Congo. The birth rate among elderly mothers was 28.7%, with a mean age of 38.6 (SD: 2.64) years. Primiparous married housewives with a university education, a history of spontaneous abortion, scarred uterus, hypertension, and PE were more concerning. Adverse obstetric outcomes include preeclampsia, pregnancy-induced hypertension, prematurity, low birth weight, and admission to the neonatal unit. The birth rate among elderly mothers was 28.70% at the Departments of Gynecology and Obstetrics at CUK and HSJ. The evolution of these birth rates among AMAs is sawtooth ranging from 12.80% in 2018 to 32.35%, passing through 31.00% in 2019, 16.4% in 2020, and 33.90% in 2021. Our birth rate figures among elderly mothers are lower than those of Debelo *et al*. in 2020 in Ethiopia [13] who found 50.0% and higher than those of Ngowa *et al*. in Cameroon in 2013 [14] and Shan *et al*. in China in 2018 [7] who found 3.70% and 7.21% respectively. The difference between these results and ours could be explained by the multicentric nature of Debelo's study with 4 maternity units and a large sample. Our results are almost similar to those of Patrick *et al*. in Kisangani (DR Congo), who found a birth rate of 27.56% among mothers of advanced maternal age [15]. The fact that our study populations belong to the same country with the same characteristics justifies this similarity.

Birth rates among mothers aged 35 and over increased from 8.6% in 1990 to 25.9% in 2012 in Japan [3]. Similar trends have been observed in other developed countries such as Manchester [9], London [10], and Australia [11]. This trend of increasing birth rates among mothers of advanced age is the same in our study where the evolution of this frequency increased from 12.80% in 2018 to 32.50% in 2022, and in our city of Kinshasa where this frequency increased from 14.68% at the General Hospital of Bumbu according to Kaka *et al*. in 2014 to ours [12]. According to Usta I *et al*. in 2008 [16] and Olusanya B *et al*. in 2012 [17], the delay in procreation and the increase in birth rates among mothers of advanced age can be attributed to several reasons: late marriage, late conception due to infertility, academic and professional opportunities, desire to have a large family, ineffective family planning sometimes non-existent and longer life expectancy. These reasons may also explain our results.

Regarding sociodemographic characteristics, in our case series, the average age of advanced mothers was 38.65 (SD: 2.64) years, with the majority of cases being housewives, married, and with a university education. According to various reports from the DR Congo Demographic and Health Surveys (DHS-DRC) in recent years, the proportion of women with university education in the DRC has increased significantly. Indeed, the frequency of women with university education increased from 1.4% in 2007 [18] to 3.7% in 2013 [19], reaching 6.6% in 2024 [20]. This explains our results. In Africa in general and in the DRC in particular, the spread of schooling with the extension of the duration of studies, family planning and today the advent of assisted medical procreation are cited among the factors explaining the postponement of pregnancy associated with the increase in the prevalence or frequency of births among AMA [5,13,21]. Indeed, it is the most qualified women who delay the first birth [22]. During studies, births are rare due to the difficulty of reconciling a life as a parent with that of a student [4,5,21]. This also explains our results. Our results are contrary to those of Shan *et al*. in China [7] who found the low level of education in more than 52% of elderly mothers living in rural areas. It is worth noting that low educational level and low socioeconomic level, and intense psychosocial stress negatively influence maternal and fetal obstetric outcomes in pregnant women: this reflects the importance of education, which had been considered as a very powerful influencing factor on perinatal outcomes [23,24].

As for the clinical profile, the average parity of mothers in AMA is 3.69 (SD: 1.96), and primiparas predominate, 43.2% of our study population. These high frequencies of primiparas in advanced age indicate that more and more women in Kinshasa are choosing to postpone their first pregnancy beyond the age of 35, an attitude also observed in several countries around the world. Indeed, similar results have been reported in the USA, where 11% of all first pregnancies concerned women aged 35 and over [25]. In Japan, the proportion of primiparous women aged 35 and over has almost tripled over the past 20 years, from 11.9% in 2000 to 29.1% in 2019 [26]. This trend towards increasing frequencies of delayed pregnancy and AMA can be attributed to several reasons: late marriage, late conception due to infertility, academic and professional opportunities, desire for a large family, ineffective family planning that sometimes does not exist, and longer life expectancy [16,17].

A history of spontaneous abortion is present in 43.10%. Our results are consistent with those of Shan *et al*. in China [7], who also found that the majority of their study population had a history of abortion (49.5%). Our results can be explained by the difficulty of reconciling life as a parent with that of a student in our study population, which mainly consisted of university students. A history of scarred uterus is present in 29.61%. According to Shan *et al*. in China [7], scarred uteri are increased due to the significantly increased risk of elective cesarean sections in older mothers. This is thought to be due to higher rates of pregnancy complications at this advanced age, the reduced number of oxytocin receptors, and the incompetent contraction capacity of the aging myometrium, making cesarean section an easy choice for physicians in the face of older mothers, and maternal demand being another undeniable and important factor nowadays. This explains our results. Several studies conducted in Asian, European, and American countries reported the cesarean section rate in older mothers ranging from 53.3% to 91.8% [11,27-31]. This explains the significant presence of scarred uteri in mothers of advanced age in our case series.

The weaknesses of our study are that it did not study maternal and fetal obstetric outcomes in mothers with AMA, and its retrospective nature does not allow us to determine the cause of this late motherhood, or include other independent factors such as the behavior of mothers in advanced age, their knowledge, and attitudes. Its strengths are that it updated the epidemiological and clinical data of mothers of advanced maternal age in our city of Kinshasa.

## Conclusion

The birth rate among advanced-age mothers is a real public health problem, with a high incidence of 28.7%. University-educated, married, and homemaker primiparous mothers with a history of spontaneous abortion, gestational hypertension, and PE, and those with a scarred uterus, are the most affected in our city of Kinshasa. Adverse obstetric outcomes include preeclampsia, pregnancy-induced hypertension, prematurity, low birth weight, and admission to the neonatal unit. Our results provide a basis for screening for various pathologies during prenatal visits in women with advanced maternal age in our city of Kinshasa, DR Congo.

### What is known about this topic

- Advanced maternal age (AMA) refers to women aged 35 years or older at the estimated date of delivery, based on the woman's physiology;
- Pregnancies among women of advanced maternal age are pregnancies at high risk of maternal and fetal morbidity and mortality;
- Updated data concerning AMA in our Kinshasa milieu does not exist.

### What this study adds

- The birth rate among elderly mothers is a real public health problem, with a high incidence of 28.7%;
- University-educated, married, and homemaker first-time mothers are most affected in the city of Kinshasa;
- Surrogate mothers with scarred uteri and a history of spontaneous abortions, HTAG, and PE are most affected in our city of Kinshasa.
